# Silencing the buzz: a new approach to population suppression of mosquitoes by feeding larvae double-stranded RNAs

**DOI:** 10.1186/s13071-015-0716-6

**Published:** 2015-02-12

**Authors:** Steve Whyard, Cassidy NG Erdelyan, Alison L Partridge, Aditi D Singh, Nigel W Beebe, Rupert Capina

**Affiliations:** Department of Biological Sciences, University of Manitoba, Winnipeg, MB Canada; School of Biological Sciences, University of Queensland, Brisbane, Australia; CSIRO Biosecurity Flagship, Brisbane, Queensland Australia

**Keywords:** Sterile insect technique, RNA interference, Testis, Spermatogenesis, *Aedes aegypti*, Dengue, Doublesex

## Abstract

**Background:**

Mosquito-borne diseases threaten over half the world’s human population, making the need for environmentally-safe mosquito population control tools critical. The sterile insect technique (SIT) is a biological control method that can reduce pest insect populations by releasing a large number of sterile males to compete with wild males for female mates to reduce the number of progeny produced. Typically, males are sterilized using radiation, but such methods can reduce their mating competitiveness. The method is also most effective if only males are produced, but this requires the development of effective sex-sorting methods. Recent efforts to use transgenic methods to produce sterile male mosquitoes have increased interest in using SIT to control some of our most serious disease vectors, but the release of genetically modified mosquitoes will undoubtedly encounter considerable delays as regulatory agencies deal with safety issues and public concerns.

**Methods:**

Testis genes in the dengue vector *Aedes aegypti* were identified using a suppression subtractive hybridization technique. Mosquito larvae were fed double-stranded RNAs (dsRNAs) that targeted both the testis genes and a female sex determination gene (*doublesex*) to induce RNA interference (RNAi) -mediated sterility and inhibition of female development. Fertility and mating competiveness of the treated males were assessed in small-scale mating competition experiments.

**Results:**

Feeding mosquito larvae dsRNAs targeting testis genes produced adult males with greatly reduced fertility; several dsRNAs produced males that were highly effective in competing for mates. RNAi-mediated knockdown of the female-specific isoform of *doublesex* was also effective in producing a highly male-biased population of mosquitoes, thereby overcoming the need to sex-sort insects before release.

**Conclusions:**

The sequence-specific gene-silencing mechanism of this RNAi technology renders it adaptable for species-specific application across numerous insect species. We envisage its use for traditional large-scale reared releases of mosquitoes and other pest insects, although the technology might also have potential for field-based control of mosquitoes where eggs deposited into a spiked larval site lead to the release of new sterile males.

## Background

Mosquitoes are the world’s most serious disease vectors, transmitting parasites and viruses that infect hundreds of millions annually. Given the deficit of effective vaccines for these diseases, our best defence has been mosquito control [1]. Broad-spectrum insecticides have been used for decades to control mosquitoes, but growing concerns about the adverse effects of these chemicals on other beneficial species, and increasing incidences of insecticide resistance amongst the vectors themselves, have prompted renewed interest in species-specific control methods [1]. The sterile insect technique (SIT) is a non-insecticidal control method that relies on the release of sterile male mosquitoes that search for and mate with wild females, preventing offspring. This approach has been used successfully to control various insect pest species [2], including the New World screwworm fly [3], the tsetse fly [4], the Mediterranean fruit fly [5], and the apple codling moth [6]. Radiation or chemosterilants are often used to sterilize males, but such treatments can decrease the insects’ ability to compete with wild males for mates, often necessitating the production of more sterile males to increase efficacy [7,8]. SIT is also generally more effective if females are not released [9], as sterile female insects can still damage crops, transmit disease, or simply distract sterile males from finding wild mates, which adds the complexity of sex-sorting to the process of an effective SIT approach.

The first applications of SIT to mosquitoes encountered mixed successes during the 1960s, 70s, and 80s [10], as it proved difficult to produce sufficient numbers of competitive sterile males to suppress natural populations. More recently, elegant transgenic approaches have generated sterile male mosquitoes for the malaria vector *Anopheles gambiae* [11,12], the Asian tiger mosquito *Aedes albopictus* [13], and the dengue vector *Aedes aegypti* [14,15]. Preliminary releases of transgenic sterile *Ae. aegypti* in Grand Cayman have demonstrated an effective reduction of these mosquitoes [16]. But while transgenic approaches have renewed interest in SIT for mosquito control, the use of genetically-modified (GM) insects will require continued effort at the technical level to develop field-competitive strains adapted for different locations, and at the regulatory level to address public concerns about the possible impacts of GM insects [17,18], particularly as mosquito movements are not restricted by borders.

Given that some countries will require considerable time to resolve these regulatory and public concerns about GM technologies, we began to develop an alternative, non-GM method that delivered both mosquito sterilization and sex sorting using RNAi technologies. RNAi is a gene-silencing mechanism achieved by delivering double-stranded RNA (dsRNA) to cells or organisms [19]. As it was already known that dsRNA could be fed to mosquitoes to induce detectable RNAi [20,21], we hypothesized that RNAi could be used to produce effective sterile male *Ae. aegypti* mosquitoes by targeting genes associated with mosquito male testes and female sex determination genes.

## Methods

### Subtractive library construction and differential screening

Testes and ovaries were dissected from 2-day old adult *Aedes aegypti* mosquitoes. mRNA was isolated from these tissues, the rest of the bodies (minus gonads), and fourth instar larvae using an Oligotex mRNA Mini Kit (Qiagen). Suppression subtractive hybridization (SSH) was used to identify genes that were preferentially expressed in *Ae. aegypti* testes relative to other tissues within the male mosquito’s body. The testis-specific subtracted library was built using a PCR-Select cDNA Subtraction kit (Clontech), using testis cDNA as the TESTER source of cDNA, and using cDNA derived from the rest of the body as the DRIVER cDNA. The SSH-specific adapters were ligated to the TESTER cDNAs and the two pools of cDNA were hybridized for the forward subtracted library. Reverse subtracted libraries were built for subsequent differential screening, where the TESTER and DRIVER designations were inversed. Amplification of hybrids corresponding to common sequences was suppressed, yielding a library enriched for differentially expressed sequences within the testes. The forward-subtracted library was ligated into the pDrive plasmid vector (Qiagen) and used to transform DH5α *E. coli* cells (Invitrogen).

The efficiency of the subtraction of the testes library was estimated using qRT-PCR by comparing the abundance of a predicted non-differentially expressed gene, *β-tubulin*, using the primers tubF (CGTCGTAGAACCGTACAAC) and tubR (CAGGCAGGTGGTAATCC). The testis-subtracted library was screened for differentially expressed ESTs using a PCR-select cDNA subtraction screening kit (Clontech). Briefly, 150 *Escherichia coli* clones were selected randomly for screening, plated, and subjected to duplicate bacterial colony lifts using standard techniques. The filters were hybridized to ^32^P-labelled probes derived from total cDNA from either the forward or reverse subtracted libraries using the PCR-Select differential screening kit (Clontech). The membranes were washed with low stringency (2× SSC, 0.5% SDS; 3×, 20 min each) and high stringency (0.2× SSC, 0.5% SDS; 3×, 20 min each) buffers at 65°C. The radiolabelled DNA was detected using a PharosFX Molecular Imaging System (Bio-Rad).

### Identification of testis genes

Plasmid DNA was recovered from selected bacterial colonies (strong signal with the forward and low signal with the reverse subtracted probe) and sequenced using Big Dye v3.1 chemistry (Applied Biosystems). DNA sequences were compared to the *Ae. aegypti* genome within the VectorBase database and predicted *Drosophila melanogaster* homologs were identified using BLAST-X against the non-redundant database at NCBI with default parameters.

Tissue and sex-specific expression of the identified genes was confirmed and quantified using qRT-PCR, using primers listed in Table 1, comparing expression of the genes’ expression levels within testes, ovaries, and adult bodies minus the excised gonads. S7 ribosomal protein (*S7rp*) gene expression was used as an internal reference to compare levels of RNAi. A single reference gene was deemed sufficient as the PCR efficiencies of the primer sets were calculated using the method of Pfaffl [22], and were found to be essentially equivalent for all genes targeted by RNAi (*bol*, *fzo*, *gas8*, *nht*, *zpg*) and for the *S7rp* reference gene, with values ranging between 95.2-98.1%. Melt curve analyses were also performed and confirmed that only a single product was amplified with each primer pair in every sample. Analysis of gene expression was performed using the 2^-ΔΔCt^ method [23], comparing expression in specific dsRNA treated samples to *gus*-dsRNA treated samples.

**Table 1:** **Primers used to amplify gene fragments for dsRNA synthesis or for qRT-PCR analysis**

**Gene**	**Forward (F); Reverse (R)**	**dsRNA primers**	**qRT-PCR primers**
AAEL001033	F	TTTCAAGCAACCGGTGACAG	ACACTTCGCTCATTCCAC
	R	TTGAGGGACGTTTTGGAAGC	ACCTTGCTGTCTCCATCC
AAEL001156	F	GCAAAACTGACCATCCTGCA	CGATGTGGACTATACGGAAAC
	R	CCGTTCTTGCAGTTTCAGCT	GGATTACTTGACGGTGCTTC
AAEL001684	F	CTGTGCCGGTTATTCAGCTC	GGTATTCGTCGGTGGTATCAG
	R	GGGATGTTGTTGATCGTCGG	GCCTCGTGTTCGGTTTCG
AAEL002084	F	TTGCTGGACGAGAAGGAAGT	AAGGAGTACGAAGAGAAGAAG
	R	ACTGAGCTGGTTGGTGAAGA	CGGAAGCGGTTCATTAGG
AAEL002275	F	AGATCGAGCACTGTTTCCGA	GTCGTTTGGTGCGGCGTTTG
	R	CGTATACATGGGCCCGATCT	CCTTGTTGTCCTCCTCATCCTTGG
AAEL003501	F	CGCAAGGATCGGAAACCAAT	CAGCAGGATACGGTCTTC
	R	CATGCTGTAGATCGGGTTGC	GAATAGGTCGGATTGTTGG
AAEL003757	F	AAAGGAGCGAAGGAGACCAA	GCGGCGGATGCGATTCTC
	R	AGGTAGTGTTTCAGGGCCTC	GACTCTTGGCGGAACGATAGC
AAEL004231	F	AGCCAAAGGAAGTACGGTCA	GAGCATCAGTCGCACATC
	R	CTTTCAGCTCTCCGATGTGC	TCCTTCTCTCGCAGTAACC
AAEL004471	F	CGCGCCAAGAAGAAGATCAA	AAGCACAACACCAGGAAC
	R	ATTTTGTCCCGCAGCATAGC	GAAACCATCAGCCAAAGC
AAEL004696	F	GATACCGAAATGTGCACGCT	CCGTGCTTGAGTTGATAC
	R	CCGGTTCTTTGTCACTGCAA	ATTGGAATCTGATGGTGAG
AAEL004939	F	TATCCTGGGCAGCTGAACTC	GAACACCACCGCCATCAC
	R	CAATGCGAGAAGGTACCGTG	CCTGCTTCTTCTTGACTTTCG
AAEL005010	F	GTTTTCGTCGGTCCGGTTAG	CGGTGGAAGTGGATTGTC
	R	TTGGCTTGGGTCTCCTTGAT	CGTTCTGATTCTTGCTGATG
AAEL005975	F	TGGACAAGGCGGAACAAAAG	GAGCAGAGATGGAGGAAC
	R	CTTGATTCGAGGCCTCAACG	CAGGCGTAACAGTCGTAG
AAEL006726	F	GCATTCCTGTTCTCGTTCCC	TGGCTTTGGTTCATTGTG
	R	TGAAGTCACATTTGGCCAGC	TATCCGATGTTGGCTTCC
AAEL006841	F	CATCGGGTGTTGCTTCTACG	ACGGTGCCTATCTGAGAAG
	R	TCAAAGTACACGTGCTGCAG	GGATGCTGATGAACGCTAC
AAEL006975	F	GCCATTTCGATGCCAAAACG	CGTCTGGTGTAGGTGCTAAGTG
	R	TCGACTGAAATCCGGGAACA	TGCTTGTTCTGCCGCTTGC
AAEL007144	F	AACACTTCAACACGTGTCGG	TTCGCATACGGAGTGTTAC
	R	TCGTACACTTCAGCGGAGTT	CCTTGTGGATGTAGTCTCG
AAEL007188	F	GCAGCGCCAATATCTGAACA	TTCGCAGTCGGATTACTTCTTC
	R	TTCCCGCTTCTTCAGGTGAT	TGGTTCTTGGTGATATTCGTAGC
AAEL007434	F	CTGTCCTCGCCCAATGAATG	TGCGTTCTGTTCATAATGGTTAC
	R	CTGCAGTAAATCTCCGCACC	GTCGGGTTTGGTTTCACTCC
AAEL007544	F	ATCGTCTATGGCCGGCTTTA	AGTTCTCCTTCCGACATC
	R	TAGCGCTATGATGTCGTCGT	GTAAGCCGCACATTCATC
AAEL007684	F	AGCGATGCAGGACGAGATTA	CGCCACCAGATGTCCTAATG
	R	CGTGGGCCAGTTTCTTATCG	CAGTTGTTCCGATTGCTTCC
AAEL008428	F	TTGGGCATGCTTCACTGATG	TGTTGGATGATGTTGTGAGATGTG
	R	ATCGTCGGAGTATCGCTGTT	ATCGTCGGAGTATCGCTGTTC
AAEL008678	F	GCCGTTTCCAGGACAACTTT	CCAGTCAGAGGGCGAATG
	R	GTAGTAATCCCGCTCTGCCT	CTTCTCCGTCAGGTCATCC
AAEL009047	F	GGAACGGTGAAATCGATGGG	CACAGAGGAGGAAGTAATTG
	R	TTCACTGCTGTCGTTGTGTG	CACTATTGGACTGCTAACG
AAEL009321	F	CAGCGACGAACCACAATGTA	GTATATGTCGCCTCGGTTC
	R	GCTTATCGCCGATGGTTACC	GGGTTGTATCGGTGTTCC
AAEL009357	F	TCAAGCAAGTGCTGGACAAC	Not completed
	R	TGCCTTCAGGTCGTTCTCTT	
AAEL009553	F	CAGTAGCTTTCCGTCCATGC	GTTGCTCTTCGTCATTATTCC
	R	GACCAGCGGATAAATCGCAG	AAATCCATTGCCATCTTTGC
AAEL010639	F	GCGCACGTTATGATGGGAAT	GCGGAGATGTTACCTTGAAG
	R	TTCCCTTGCATCACATCCCT	GAAACTACCTGGACCTCTGG
AAEL011098	F	TTGGCGAAATTCTGCAAGCT	ACTCATACACTCGTTTCAAG
	R	AGCCGAAACGTTTGCTTCAT	TCCATATCCGAAGCACTC
AAEL011310	F	GGATATGAGACCCGAACCGT	GAAGATGCCGAGCGAGAG
	R	GTTTTCTTCGGTGCCTTCGT	GACCGACCTGGATGGATTC
AAEL012096	F	AATACCAGCACGCTCTCTCA	Not completed
	R	ATTGCATCGGTGGCAAGTTT	
AAEL012446	F	GCTACTTGGATTTGGGCGAC	CGGAGCCAAGGAGGTCATC
	R	ATCGCTTCGGACAGGATGAT	ACAGCAGCAGAAGCAGAGG
AAEL013621	F	TTTGAACCCGGAAAAGGCAG	CCAGAGCAACCGAGAGTATG
	R	TTCGACGAAATCCTCCCACA	CGACGAAATCCTCCCACAG
AAEL013723	F	GATGAAACTGCCGCCACTAG	TTAAGATTGTCACCTTCACC
	R	TTGCCGAACCGTTGGAAAAT	CGTTGTAGATGTTCTGTCC
AAEL013737	F	AAGACTTGGGAAGAGGACGG	TGTAGTTCGGTATCGTTCGG
	R	TTCTCTAGCAGCTGGATCCG	TCGGCATTCCTTCGTTCG
AAEL014067	F	TTGCTCATACGCTCCATTGC	ACAACAGAGCCTAAGACTATC
	R	TTGCTCCTGAACGGTGAGAT	CGACAATCATATTCTCACAGC
AAEL014408	F	GACCGATCCTGCAAAAGTCC	TGGACGATAATGCTCAAC
	R	TTTGCTCCTGGGTGTAGAGG	GAGGCGAATGGAGTTATG

### Isolation of the *dsx*^F^ gene fragment in *Ae. aegypti*

The two primers pairs – dsxf1-for (GGTCAAGCCGTGGTCAATGAAT) and dsxf1-rev (CAACATTCTCCGCGCACAGG), and dsxf2-for (GCAAATGCTGTTTAACGATAATAG) and dsxf2-rev (CGGAGCCGTTTGGCAACGG)– were used to amplify portions of the two female-specific exons of the *dsx*^F^ transcript (GenBank:DQ440532, GenBank:DQ440533). These two PCR products were subsequently used as templates to prepare dsRNAs by *in vitro* transcription, and were used in equimolar concentrations for dsRNA soaking or were cloned into pL4440 plasmids, to be used to transform *E. coli*, for bacterial feeding, as described below.

### Injecting dsRNA into mosquito pupae

Total RNA was extracted from late pupae and early adult male *Ae. aegypti*, using QIAshredders (Qiagen) to homogenize tissues and an RNeasy RNA extraction kit (Qiagen). RNA was treated with amplification grade DNase I (Invitrogen) and 1 μg was used to synthesize cDNA using a First Strand cDNA Synthesis kit (Invitrogen). The cDNA served as template DNA for PCR amplification of gene fragments ranging in size from 260 to 380 bp in length, using the primers listed in Table 1. The gene fragments were subcloned into the cloning vector pDrive (Qiagen), and later excised from pDrive using either *ApaI* and *PstI* or *MluI* and *NotI* restriction enzymes, then ligated into a similarly-digested plasmid pL4440, a vector possessing convergent T7 promoters (kindly provided by Andrew Fire, Stanford University). A 401 bp fragment of the β*-glucuronidase* (*gus*) gene, a bacterial gene specific to *E. coli*, was amplified by PCR from the pBacPAK8-GUS plasmid (Clontech) using the following primers: GusF (CCCTTACGCTGAAGAGATGC) and GusR (GGCACAGCACATCAAAGAGA). The PCR product was cloned into the dsRNA transcription plasmid pL4440, as described above, to be used as a negative control. DNA templates for *in vitro* transcription of each of the gene fragments in pL4440 were PCR-amplified using the following pL4440-specific primers: pL4440F (ACCTGGCTTATCGAA) and pL4440R (TAAAACGACGGCCAGT). PCR products were purified using a QIAquick PCR purification kit (Qiagen). The MEGAscript RNAi kit (Ambion) was then used for *in vitro* transcription and purification of dsRNAs. DsRNAs were diluted to 0.1 mg/ml in 20 mM phosphate, pH 7, and each pupa was injected with 50 nl. To assess for RNAi, insects were allowed to develop until 3-days post-eclosion and RNA was extracted and subjected to qRT-PCR analyses as described above.

### Introducing dsRNA to mosquito larvae

a) DsRNA soaking method: Mosquito larvae (from 1^st^ to 4^th^ instars) were soaked in two concentrations of *in vitro*-transcribed dsRNA (0.02 and 0.2 mg/ml dsRNA) in dechlorinated tap water for 1 h/day, for 6 days, and were returned to their feeding trays after each dsRNA exposure. b) Feeding insect dsRNA-expressing bacteria: The pL4440 plasmids containing the mosquito genes were used to transform HT115(DE3) *E. coli* cells and dsRNA production was induced by growing the liquid cultures of Luria Broth supplemented with 50 μg/ml ampicillin and 0.4 mM IPTG. Once the cultures had reached an OD of 0.7-0.8, the cells were pelleted by centrifugation and mixed with 1% LB-agar containing ampicillin and IPTG and 1 g of finely ground Tetramin fish flakes, cooled to 42°C to ensure the bacteria were not heat killed. The agar bacteria mixture was plated to a thickness of 5 mm, cooled, and then cubed into 5 mm cubes, to be fed to mosquito larvae. To feed heat-killed bacteria in the same mixture, following pelleting, the bacteria were exposed to 70°C for 1 h, and then mixed with the agar-Tetramin mixture and plated and cubed. To check that the bacteria were heat killed, 2 μl of both heat-killed and non-heat killed bacteria were separately mixed with 50 μl LB, and spread on LB plates, to check for bacterial growth. The agar cubes for mosquito feeding were stored at 4°C for up to 1 week before use. Mosquito larvae were raised in densities of 1 larva/2 ml water (groups of 10 larvae in 20 ml) and provided single cubes of bacteria-containing agar on a daily basis. As pupae developed, they were transferred to individual vials to await eclosion and sex-sorting.

### Mosquito fertility assays

Individual 3-day old dsRNA-treated mosquitoes were provided virgin mates in 25 ml plastic vials for 2 days. Mosquitoes were provided 10% sucrose *ad libitum*, and to assess fertility and fecundity, females were provided blood meals (derived from rats) 2 days post-mating, and eggs were collected on moistened paper towels placed within the vials for between a week and 10 days post-blood meal. Eggs were incubated at 25°C for 4 days, and then immersed in water to assess hatch rates and calculate fecundity. If no eggs were produced, or all eggs failed to hatch, the dsRNA-treated insect was considered sterile.

For small population mating competitions involving only 20 mosquitoes, 500 ml glass jars with screen-covered lids were used, while mating competitions using 200 mosquitoes were conducted in larger (45 X 45 x 45 cm) screened cages. All sterility and fecundity values for dsRNA-treated insects were expressed as percentages, relative to negative control animals, which were considered as 0% sterile, 100% fecund.

### Statistical analysis

For every experiment, sample size was determined empirically (preliminary experiments were performed) to ensure that the desired statistical power could be achieved. The values are expressed as mean ± s.e.m. The error bars (s.e.m.) shown for the results were derived from biological replicates, not technical replicates. Sterility and fecundity values that were expressed as percentages, relative to the values obtained from negative control treatments, were arcsine transformed to produce values that displayed a normal distribution and comparable variance. Significant differences between two groups were then evaluated using a two-tailed, unpaired *t*-test.

## Results

### Identification of testes-specific genes

To test whether RNAi could be used to develop an effective SIT technology, we used a suppression subtractive hybridization technique [24] to identify genes that were preferentially or exclusively expressed in *Ae. aegypti* testes. A testis-specific cDNA library was prepared by first hybridizing testis cDNA with cDNA derived from the rest of the body. Amplification of hybrids corresponding to common sequences was suppressed, yielding a library enriched for differentially expressed sequences within the testes. A total of 37 genes were identified that were predominantly or exclusively expressed in male *Ae. aegypti* testes. Reverse transcriptase-PCR (RT-PCR) confirmed that all 37 genes were expressed in the testes, with 15 genes showing no evidence of expression in larval stages, other adult tissues, nor in female ovaries. Six genes were expressed in both testes and ovaries, but not in other adult tissues. Homologs for all but a few of the genes were identified in *Drosophila melanogaster*, and many were similarly expressed in either testes or ovaries (Table 2), providing more than a dozen target genes to use.

**Table 2:** **Genes expressed predominantly or strictly in the testis of** ***Ae. aegypti***

***Aedes aegypti*** **accession #**	***D. melanogaster*** **homolog**	**Testis-specific?**	**Expressed in larvae?**	**Ovary-specific?**
AAEL001033	CG8208 (*MDB-like*)	No	Yes	Yes
AAEL001156	CG5280	Yes	No	No
AAEL001684	CG4727 (*bol*)	Yes	No	No
AAEL002084	CG14220	Yes	No	Yes
AAEL002275	CG3565	Yes	No	No
AAEL003501	CG10252	Yes	Yes	No
AAEL003757	CG4434	No	Yes	No
AAEL004231	CG14271 (*Gas8*)	Yes	No	No
AAEL004471	CG4568 (*fzo*)	Yes	No	No
AAEL004696	CG5737	No	Yes	No
AAEL004939	CG32396 (β*-tub*)	Yes	No	No
AAEL005010	CG14305	Yes	No	No
AAEL005975	CG15259 (*nht*)	Yes	No	No
AAEL006726	CG6647 (*zpg*)	Yes	No	Yes
AAEL006841	-	Yes	No	Yes
AAEL006975	CG18369 (*S-Lap5*)	Yes	No	No
AAEL007144	CG12423 (*klhl10*)	No	No	No
AAEL007188	CG17083	Yes	No	No
AAEL007434	-	Yes	No	No
AAEL007544	CG10895 (*lok*)	Yes	No	Yes
AAEL007684	CG4767 (*tek*)	No	Yes	No
AAEL008428	CG10841	No	No	No
AAEL008678	CG18190	No	Yes	Yes
AAEL009047	CG8819 (*achi*)	No	Yes	Yes
AAEL009321	-	No	Yes	Yes
AAEL009357	CG2146	No	Yes	Yes
AAEL009553	CG12813	No	Yes	No
AAEL010639	CG5458	Yes	No	No
AAEL011098	CG8362	Yes	No	Yes
AAEL011310	CG9313	Yes	No	No
AAEL012096	CG18472	Yes	No	No
AAEL012446	CG6303	No	Yes	Yes
AAEL013621	CG17564	Yes	Yes	No
AAEL013723	CG31000 (*heph*)	Yes	Yes	Yes
AAEL013737	CG6971	Yes	No	No
AAEL014067	CG5048	Yes	Yes	No
AAEL014408	CG4965 (*twe*)	Yes	No	Yes

Genes were identified in a SSH screen, selectively amplifying genes expressed in testes relative to the rest of the male’s body. cDNA was extracted from dissected male testes, the male body minus testes, 4^th^ instar larvae, dissected female ovaries, and female bodies minus ovaries, and RT-PCR was then used to assess whether the genes were 1) testis-specific (i.e. in testes, but not other adult tissues); 2) expressed in larvae; and 3) ovary specific (i.e. in ovaries, but not other female tissues). Genes are ordered in ascending VectorBase (www.vectorbase.org) accession numbers.

### Development of RNAi-induced sterile and low fertility males

**Figure 1:**
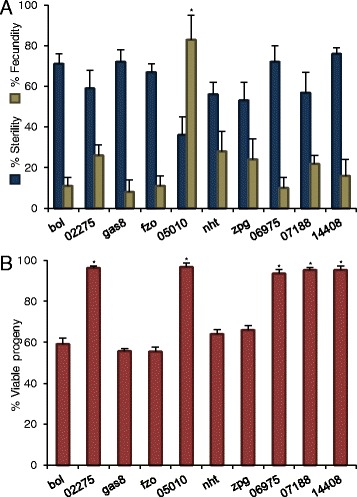
**RNAi screen for mosquito SIT target genes.** Ten gonad-specific dsRNAs were injected into pupae and 20 males were tested for fertility, fecundity, and mating competitiveness. **A**, Percentages of sterile males (blue bars) and fecundity of remaining, semi-fertile males (green bars), relative to negative control males, are indicated. **B**, Mating competitiveness of dsRNA-injected males was assessed in small (5 dsRNA males, 5 untreated males, 10 females) mating competitions by calculating percent viable progeny, relative to negative controls. Treatments not differing from the controls are marked (*; student t-test, P > 0.05). Error bars indicate the s.e.m. of three independent trials.

A subset of 10 genes was selected for RNAi-mediated knockdown to determine if they would render males sterile. In addition to sterilizing the males, the knockdown of the target gene should not adversely affect the male’s ability and willingness to mate, as males must still compete effectively with wild, fertile males. For this reason, we selected genes that were not expressed in other tissues of the male, thus avoiding any adverse effects on mating behaviors or physiologies. DsRNAs targeting the 10 genes were injected into pupae, and subsequent quantitative RT-PCR (qRT-PCR) confirmed that each of the 10 transcripts were knocked down by between 70-95% within the adult males four days’ post-injection. The dsRNA-injected males were then mated with virgin females, and most (9/10) of the dsRNAs induced complete sterility in more than half of the injected males, and substantially reduced fecundity in the others, relative to either non-injected males or to males injected with an *E. coli*-specific dsRNA (Figure 1A).

Mating competiveness of the sterilized males was also assessed by combining them with untreated males and virgin females in small population cages. When the number of progeny was scored, five dsRNAs both induced male sterility and caused significant reductions (>75%) in the number of viable progeny produced (Figure 1B). These particular dsRNAs all targeted mosquito genes that had homologs in *D. melanogaster* and were known to be involved in spermatogenesis: *boule* (*bol*); *fuzzy onions* (*fzo*); *growth arrest specific protein 8* (*gas8*); *no-hitter* (*nht*); and *zero population growth* (*zpg*). One of the genes, *zpg*, had previously been identified as important for male fertility in the malaria vector *An. gambiae*: dsRNA targeting *zpg* transcripts injected into *An. gambiae* embryos produced males that lacked sperm, but they could still transfer male accessory gland fluids to the females to induce female post-mating behaviors, including oviposition of unfertilized eggs [25].

The other five dsRNAs tested in this screen produced sterile or partially sterile males that were not strongly competitive, allowing the females to continue to mate with fertile males. While these dsRNAs had targeted genes that were apparently expressed exclusively in the testis, their knockdown may nevertheless have affected male mating performance by reducing the male’s mating activity, sperm transfer, or transfer of male accessory gland proteins – some of which can affect female mating behavior and fecundity [26].

**Figure 2:**
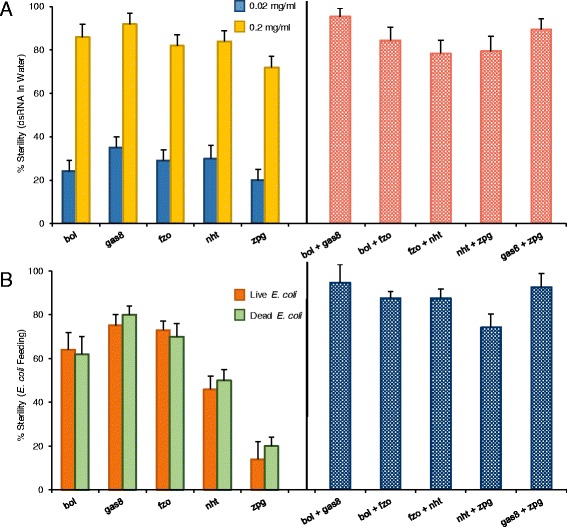
**Feeding dsRNAs to larvae induces adult male sterility or low fertility. A**, 100 larvae (from 1st to 4th instars) were soaked for 1 h each day for 6 consecutive days in two concentrations of five dsRNAs (left panel) or in mixtures of two dsRNAs (each 0.01 mg/ml, right panel); the percentage of sterile males, relative to untreated controls, are indicated. **B**, 100 larvae were fed either live or heat-killed bacteria expressing one dsRNA (left panel) or mixtures of live *E. coli* expressing two dsRNAs (right panel), and the percent of sterile males, relative to untreated males, was assessed. Error bars indicate the s.e.m. of five independent trials. All dsRNA treatments induced significant reductions in fertility (ANOVA, P < 0.01).

The five spermatogenesis-specific dsRNAs were subsequently administered to mosquito larvae by soaking larvae in dsRNA solutions (at two concentrations: 0.02 mg/ml and 0.2 mg/ml) for one hour each day for six days. At the higher concentration, these brief exposures to the dsRNAs sufficiently induced sterility in most (72-92%) of the males (Figure 2A). The small number of fertile males had severely reduced fecundity. The lower concentration of dsRNAs produced fewer sterile males (20-35%) but, encouragingly, by mixing low concentrations of different dsRNAs together, sterility frequencies could be enhanced to nearly 100% (Figure 2A). This suggests that combinations of dsRNAs may permit lower concentrations of dsRNAs to be used for sterile male production. In these dsRNA soaking treatments, the dsRNA likely entered the insects by ingestion, although entry through other routes (e.g. cuticular penetration) cannot be excluded. Nevertheless, RNAi clearly spread beyond the initial entry points to reach the testes, and this relatively simple “bathing”, or soaking, technique underscores one of the many advantages of this new transformative approach to SIT.

### Bacterial dsRNA mosquito larval treatment

Mosquito larvae were fed *E. coli* bacteria expressing dsRNAs, in a manner similar to that used to feed *Caenorhabditis elegans* nematodes [27]. Both live and heat-killed *E. coli* were embedded into agar cubes containing finely ground larval diet, on which the mosquito larvae grazed until pupation. Three of the five *E. coli* strains expressing dsRNA sterilized more than 60% of the males, and by combining different dsRNA-expressing *E. coli* strains, up to 92% sterility could be achieved (Figure 2B). No significant loss of potency of the RNAi effect occurred when the bacteria were heat-killed (Figure 2B).

### Biasing emerging adults towards males

To produce a male-biased population of mosquitoes, and obviate the need for later sex-sorting, we also attempted to silence a female differentiation gene. In many insects, the *doublesex* (*dsx*) gene is alternatively spliced in males and females, with the female splice variant of *dsx* producing DSX^F^, which acts as a transcription factor that regulates many female-specific genes throughout an insect’s development and in tissues that display sexual dimorphic development [28]. RNAi-mediated knockdown of the female splice variant of *dsx* (*dsx*^F^) was achieved by either soaking the mosquito larvae in dsRNA, or by feeding the larvae *E. coli* expressing dsRNA that targeted the two female-specific exons of *Ae. aegypti dsx* [29]. Both dsRNA delivery methods resulted in a strongly male-biased population of insects (up to 97% males; Table 3). The fact that the number of dsRNA-treated larvae that developed into adults was virtually halved relative to the negative controls, with no significant increase in males (Chi-square test, P > 0.05), suggests that the vast majority of RNAi-affected females simply failed to develop, rather than having undergone a sex-reversal due to the lack of *dsx*^F^. Although a complete lack of females remains preferable for any SIT approach, the few extant females produced were either sterile, or failed to blood-feed, mate or survive, and hence, should not compromise the efficacy of this technique.

**Table 3:** **Treatment of mosquito larvae with** ***dsx*** ^**F**^ **-dsRNA reduced production of females and all were sterile**

**dsRNA**	**dsRNA delivery**	**# Larvae treated** ^**3**^	**Females/males that developed**	**# Females that blood fed**	**# Females that produced progeny** ^**4**^
gus	Daily soakings^1^	420	207/200	162	139
dsx^F^	Daily soakings^1^	440	6/242	0	0
gus	Larval feeding^2^	445	238/187	194	172
dsx^F^	Larval feeding^2^	460	7/241	1	0

^1^Larvae (1^st^-4^th^ instars) were soaked for 1 h/day for 6 days in 0.2 mg/ml dsRNA.

^2^Larvae were fed heat-killed *E.coli* continuously.

^3^Mixed sexes of larvae were treated.

^4^Females were provided 2 males for a period of one week, offered blood meals, and hatching of eggs monitored.

### Mating competitions with RNAi-induced sterile and low fertility males

**Figure 3:**
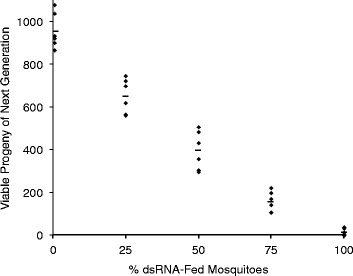
**Seeding populations with dsRNA-treated mosquitoes reduces production of progeny.** Larvae (first to fourth instars) were soaked daily for 1 h for 6 consecutive days in a mixture of *dsx* ^F^ and *gas8*-dsRNAs (0.2 mg/ml each). The adults arising from these treatments were mixed at different proportions with untreated males (100 males in total), and were mixed with 100 virgin females. The number of viable progeny from each mating cage was assessed 10 days later. Each point represents a single cage experiment (i.e. 6 replicates for each treatment), with the average number of progeny indicated (horizontal lines). Progressive increases in sterile male seeding density produced significantly greater reductions in the number of progeny produced (ANOVA, P < 0.01).

When dsRNA targeting male-specific *gas8* was combined with dsRNA targeting *dsx*^F^ and fed to *Ae. aegypti* larvae, the mosquitoes that developed were almost entirely males (96%), and the vast majority of these (96%) were sterile. The remaining 4% of males were only partially fertile, producing, on average, less than 20% of the progeny produced by fertile males. We then mixed the dual dsRNA-treated mosquitoes in varying proportions with untreated male mosquitoes in population cages holding 100 untreated females. These mating competition experiments suggested that the dsRNA-treated males were very effective competitors, as significant reductions in progeny were observed when the populations were seeded with 25% dsRNA-treated males (ANOVA, P < 0.01) (Figure 3); at 50% dsRNA-treated males, 50% reduction in progeny was observed. At higher sterile male seeding densities, very strong population suppression was observed, ranging from 95 to 100%. Even without completely eliminating the few contaminating females from the dsRNA-treated insects, the sterilized males were clearly good competitors and proved effective at reducing mosquito populations in these cage settings.

## Discussion

This study demonstrates that it is possible to generate non-transgenic, non-radiation sterile or low fertility male mosquitoes that efficiently mate with wild females, thus preventing the production of offspring and reducing the population size. Using the dengue vector *Ae. aegypti* as a model system, dsRNAs were introduced to mosquito larvae to induce an RNAi-mediated knockdown of male fertility genes and a female sex determination gene, overcoming the need to sex-sort insects to produce a male-biased, substantially sterile adult population.

These outcomes are both essential and exciting in terms of designing efficient SIT approaches in the future. They build on previous mosquito population suppression techniques, which release sterile male insects to seek out and mate with females, a proven biological control method for insects [14,30]. The RNAi-based approach, however, offers some unique adaptations to conventional SIT, including the potential for species-specificity of the dsRNA-based sterilizing molecules, avoiding the debilitating effects of radiation, and eliminating the need to sex-sort the insects before release. The RNAi effect following feeding on dsRNAs was remarkably robust and persistent throughout the development of the mosquitoes – a phenomenon that has been observed in a variety of insect species [31-33]. Exposure of the larvae to the testis-specific dsRNAs before and during early testis development was sufficient to cause adult sterility or very low fertility. Similarly, exposure to the *dsx*^F^ dsRNA was sufficient to inhibit complete development of females. While *dsx* is absolutely required for the early stages of sex differentiation during embryonic development, it is also required for proper differentiation and development of the adults [28]. Homologs for *dsx* have already been identified in *Aedes* and *Anopheles* mosquitoes [34,35], and we anticipate that many of the genes identified as effective dsRNA-sterilizing targets by this study will have homologs in other insects. In our estimation, there is considerable potential to apply this novel technology to other insects amenable to SIT approaches, including important agricultural and human pathogen vectors.

This RNAi method could be applied to both mass-reared insects and on insects collected from the field, which could be used to supplement laboratory-bred populations to counter possible declines in male vigor due to chronic inbreeding [36,37], or to minimize assortative mating behaviors that females could exhibit when exposed to mass-reared males [38]. While it is anticipated that RNAi technology could be easily adapted for insect mass-rearing facilities, the method also has the potential for field-based control via administration of either dsRNA-baited food sources or artificial larval environments. These approaches would see only competitive sterile or low fertility males emerge from eggs oviposited into the larval site and could potentially drive a local population to extinction.

In terms of the economics of this approach, our method of producing dsRNAs by *in vitro* transcription would be prohibitively expensive to treat large numbers of mosquitoes (~$200 to produce 1 mg of dsRNA to treat 100 larvae for a single experimental trial), but recent advances in large-scale (kg quantities) production of dsRNAs [39] will greatly reduce costs. Production of dsRNA in bacteria [40] or other microorganisms [41] is a very cheap method of producing dsRNA and interestingly, heat-killed bacteria were just as effective as live bacteria at inducing RNAi in the mosquitoes. From a regulatory perspective, heat-killed bacteria may prove safer in terms of possible distribution of dsRNA into natural or artificially-constructed mosquito larval sites. For some species, the use of RNA microcarriers, such as liposomes [20] or nanoparticles [21] might be required to facilitate uptake of the dsRNA and/or stabilize the dsRNA in the larval environment.

While many of the genes identified in this study are shared with other insects, RNAi has an in-built sequence-specificity [42,43], which, with careful design of the dsRNAs through knowledge of the gene sequences of sympatric species, could be developed to specifically target pathogen-transmission or pest species without impacting non-target species. This ability enhances the appeal of this new insect control technique, addressing yet another point of concern in earlier approaches in terms of impacts on other species in general and on beneficial species in particular.

## Conclusions

Releasing sterile male insects to mate with wild females is an established method of insect population control. Previous technologies have been hampered by their need for radiation (that reduces male fitness) or genetic modification (with its risks and regulatory issues) to generate the sterile males. Here, we demonstrate production of non-radiated, non-transgenic sterile or extremely low fertility males of the dengue vector *Ae. aegypti*. By feeding dsRNA to the insects at larval stages, sperm production in adult males can be halted (producing fit substantially-sterile males) with a bias towards males emerging as adults, which avoids sex sorting. Our species-specific sequenced-based targets are common in other insects and may thus open a new industry in vector and pest control.

## Abbreviations

dsRNA: Double-stranded RNA
RNAi: RNA interference
GM: Genetically-modified
SIT: Sterile insect technique
*dsx*: *Doublesex*
*bol*: *Boule*
*fzo*: *Fuzzy onions*
*gas8*: *Growth arrest specific protein 8*
*nht*: *No-hitter*
*zpg*: *Zero population growth*
qRT-PCR: Quantitative reverse transcriptase-polymerase chain reaction
